# *Malassezia restricta as* an unexpected cause of infectious osteomyelitis diagnosed by metagenomic sequencing: a case report and literature review

**DOI:** 10.1186/s12879-024-09512-9

**Published:** 2024-06-26

**Authors:** Guoxuan Peng, Yuan Lin, Qiang Zou, Hongcheng Peng, Anyi Lei, Xu Zou, Zhe Xu, Hong Sun, Xu Ning, Mingzhi Huang

**Affiliations:** 1 Department of Orthopaedics, Affiliated Hospital of Guizhou Medical University, Guiyang, 550001 Guizhou China; 2 School of Clinical Medicine, Affiliated Hospital of Guizhou Medical University, Guiyang, 550001 Guizhou China; 3 Guihang Guiyang Hospital, Guiyang, 550006 Guizhou China

**Keywords:** *Malassezia. Restricta*, Metagenomics, Vertebral osteomyelitis, Infection

## Abstract

**Background:**

*Malassezia restricta*, a lipophilic and lipodependent yeast belonging to the basidiomycetes group, is an opportunistic fungal pathogen associated with various skin diseases, including seborrheic dermatitis and dandruff. Typically, *Malassezia* infection in neonates manifests as fungemia or hematogenous dissemination to the bone or lungs. However, vertebral osteomyelitis caused by these fungi is rarely reported owing to non-specific clinical presentations and laboratory/imaging findings. The Pathogen Metagenomics Sequencing (PMseq) technique enables direct high-throughput sequencing of infected specimens, facilitating the rapid and accurate detection of all microorganisms in clinical samples through comprehensive reports.

**Case presentation:**

A 52-year-old male was admitted to our hospital on July 20, 2022 with a 3-month history of ambulatory difficulties and localized low back pain. Magnetic Resonance Imaging (MRI) examination of the spinal column revealed irregular bone destruction affecting the L2, L3, and L5 vertebral bodies. Additionally, low T1 and high T2 intensity lesions were observed at the intervertebral discs between L3 and L5. The presumptive diagnosis of tuberculous spondylitis was made based on the imaging findings, despite negative results in all mycobacterium tests. However, the patient exhibited no improvement after receiving regular anti-tuberculosis treatment for 3 months. Subsequent MRI revealed an expansive abnormal signal within the vertebral body, leading to progressive bone destruction. The absence of spinal tuberculosis or other infective microorganisms was confirmed through culture from blood and pathological tissue from the L4 vertebral body. Subsequently, PMseq was performed on the specimens, revealing *M. restricta* as the predominant pathogen with the highest relative abundance value. The pathological examination revealed the presence of fungal mycelium in the L4 vertebral body, with positive findings on periodic Schiff-methenamine and periodic acid-Schiff staining. The anti-tuberculosis treatment was discontinued, and an antifungal combination of fluconazole and voriconazole was administered. All symptoms were resolved after 7 consecutive months of treatment, and the patient was able to ambulate autonomously. Vertebral lesions were reduced on MRI during the 13-month follow-up.

**Conclusions:**

*M. restricta* is not a commonly recognized pathogen associated with infectious vertebral osteomyelitis. However, PMseq can aid in diagnosis, timely treatment, and decision making for some non-specific infectious diseases.

## Background

The etiology of spinal osteomyelitis encompasses a wide range of pathogens, including bacteria, viruses, fungi, *Mycobacterium tuberculosis*, and parasites [1]. Although tuberculous osteomyelitis is the most prevalent spinal infection, fungal infections of the spine, particularly in immunocompromised or critically ill patients, can also be caused by *Candida*, *Cryptococcus*, *Aspergillus* species, and other molds [2–4]. In the case of the other fungi, spinal involvement typically occurs owing to hematogenous or direct spread of organisms from an initial pulmonary source of infection. Moreover, involvement of the vertebral bodies can result in severe lumbar pain, impaired ambulation, vertebral compression fractures, and pronounced spinal deformity [5]. *Malassezia restricta* is an opportunistic fungal pathogen associated with a range of dermatological conditions, including seborrheic dermatitis and dandruff [6, 7]. To date, occurrences of vertebral infection caused by *M. restricta* are exceedingly uncommon. Recently, Limon et al. [8] demonstrated that *M. restricta* elicits innate inflammatory responses largely linked to the presence of an inflammatory bowel disease-associated polymorphism through the signaling adaptor gene of CARD9 in Crohn’s disease. Owing to the non-specific clinical manifestations and imaging characteristics of fungal vertebral osteomyelitis, accurate diagnosis is typically challenging [9]. Additionally, a minimum of 3 days of culture at temperatures ranging from 23 to 28 °C is typically required for *Malassezia* growth. It should be noted that false-negative results may occur in the culture, serum 1-3-β-D-glucan test, and serum galactomannan (GM) test [10].

Pathogen Metagenomics Sequencing (PMseq) involves direct high-throughput sequencing of infected specimens, followed by comparison with microbial-specific databases and intelligent algorithm analysis. This approach enables the identification of species information for suspected pathogenic microorganisms, facilitation of rapid and accurate detection of challenging and critical infections, and comprehensive and in-depth genomics analysis of total DNA [11, 12]. Moreover, PMseq demonstrates a significantly reduced turnaround time of approximately 24 h encompassing sampling, nucleic acid extraction, library sequencing, data processing, and reporting in comparison to conventional methods. Consequently, it enables prompt pathogen identification in instances of inexplicable illnesses [13]. We employed the PMseq method to successfully identify *M. restricta* as the probable causative agent of vertebral osteomyelitis in a patient who was initially diagnosed with tuberculosis (TB) but did not respond to regular anti-TB agents after 3 months of treatment. Furthermore, the implementation of antifungal therapy has demonstrated significant improvements in patient outcomes, encompassing alleviation of low back pain, restoration of ambulatory capacity, and mitigation of vertebral lesions. Herein, we present a case of fungal vertebral osteomyelitis potentially caused by *M. restricta* in an immunocompetent patient.

## Case presentation

**Fig. 1A:**
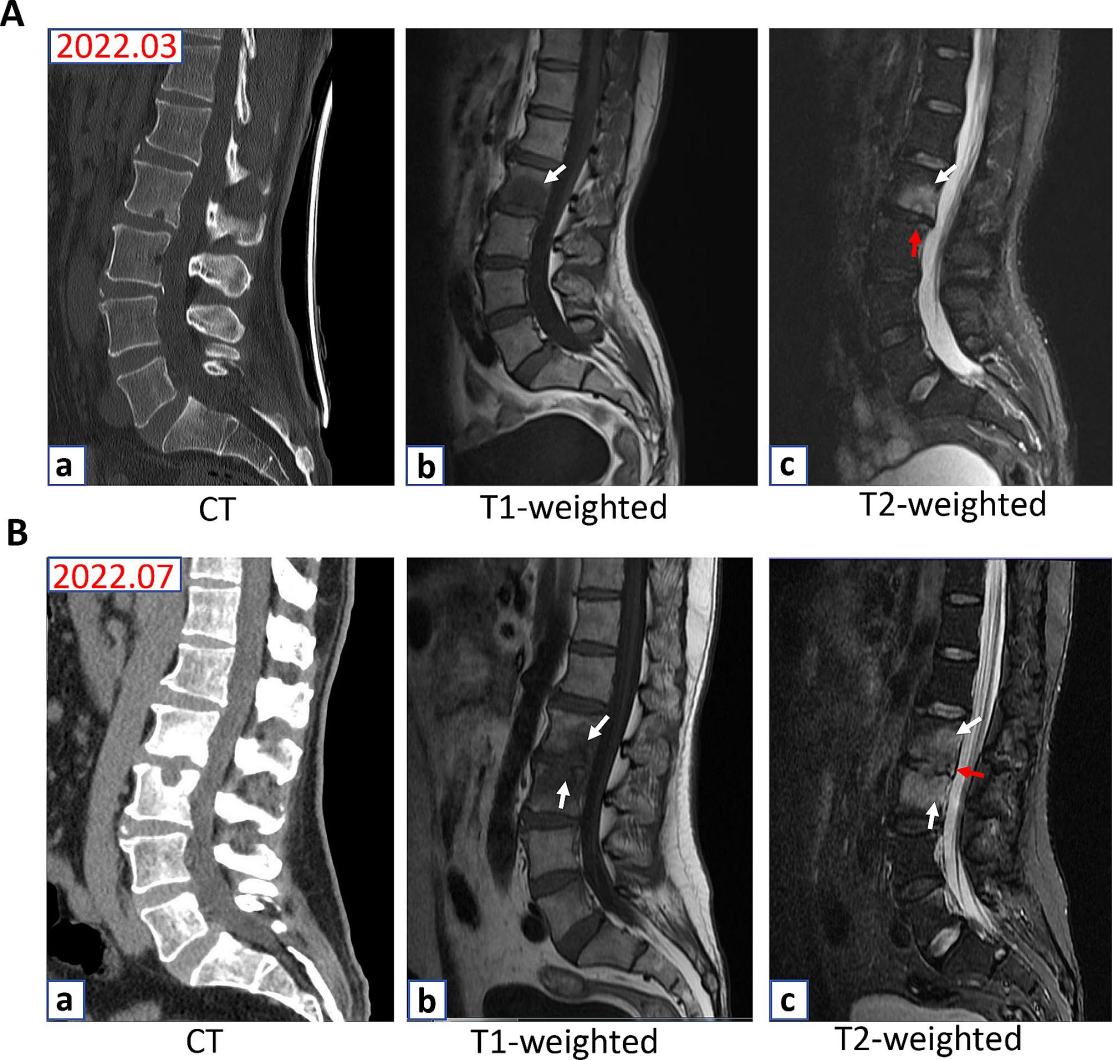
CT showed an irregular bone destruction at the L2 vertebral body (**a**). MRI showed irregular bone destruction at the L2 vertebral body endplate (white arrow) with a T2-weighted image (c) and a lesion with low T1 (**b**) and high T2 intensity (**c**) at the L2 vertebral body and L2–L3 intervertebral disc (red arrows) on March, 2022. Fig. **1B** CT showed a significant irregular bone destruction at the L2 and L3 vertebral body (**a**). MRI showed irregular bone destruction at the L2 and L3 vertebral body endplate (white arrow) with a T2-weighted image (**c**) and a lesion with low T1 (**b**) and high T2 intensity (**c**) at the L2 and L3 vertebral body and L2–L3 intervertebral disc (red arrows) on July, 2022

A 52-year-old male presented with a 3-month history of ambulatory difficulties and lumbar pain. Magnetic Resonance Imaging (MRI) examination of the spine revealed irregular bone destruction affecting the L2, L3, and L5 vertebral bodies. Additionally, low T1 and high T2 intensity lesions were observed at the intervertebral discs between L3 and L5 3 months earlier (Fig. 1A). The primary diagnosis was considered to be spinal TB; however, the T-SPOT.TB test and acid-fast staining of blood yielded negative results, whereas computed tomography (CT) did not reveal any signs of pulmonary tuberculosis. Therefore, the patient underwent a CT-guided percutaneous biopsy of spinal lesions owing to an unidentified diagnosis based on clinical manifestations and laboratory/imaging findings. Furthermore, both culture and pathological examination of vertebral specimens failed to find any evidence of pathogen infection. Despite the absence of conclusive evidence supporting *Mycobacterium. tuberculosis* infection, the potential for spinal TB remained substantial based on its imaging characteristics. Therefore, with the consent of family members, the patient underwent anti-TB treatment at a local hospital. However, despite receiving oral anti-TB medications for a duration of 3 months, there was no improvement in his symptoms; on the contrary, his back pain had notably intensified over the past month. On July 20, 2022, the patient was admitted to our hospital with recurrent severe low back pain (visual analog scale (VAS) score of 9 out of 10 points) and difficulty standing for 1 week. Laboratory investigations yielded the following results: serum C-reactive protein (CRP), 8.41 mg/L (normal value: < 5 mg/L); urine kappa-light chain (K-LC), 13.5 mg/L (normal value: 0.00 − 7.13 mg/L); erythrocyte sedimentation rate (ESR), 6.0 mm/h (normal value: 0–21 mm/h); Interleukin-6 (IL-6), 6.61 pg/ml (normal value: 0 − 7 pg/ml); procalcitonin, 0.03 ng/ml (normal value:<0.15 ng/l); serum 1-3-β-D-glucan,<37.5 pg/ml (normal value: 0 − 70 pg/ml); serum GM<0.008 (normal value:<0.5 S/CO); serum LAMBDA-light chain (λ-LC)<3.94 mg/L (normal value: 0.00 − 3.94 mg/L); HIV (negative). The first–third doses of COVID-19 vaccine were administered in August, September, and October 2021, respectively. Furthermore, the patient exhibited a state of overall good health with no known allergies, other immune deficiencies, cardiovascular disorders, or diabetes mellitus. Additionally, the patient had no skin abnormality including atopic dermatitis, psoriasis, dermatophytosis, and tinea cruris. However, the patient has not received any acupuncture or moxibustion treatment before the onset of the disease. Positron emission tomography-CT scans showed metabolically active glands and inflammatory uptake in the destructive vertebral bodies of L2, L3, and L5. Repeat MRI showed an expansive presence of abnormal signals in the vertebral body, resulting in progressive bone destruction compared to previous MRI findings (Fig. 1B). After obtaining informed consent from family members, a CT-guided percutaneous biopsy procedure was conducted to obtain specimens of the spinal lesions located at the L4 vertebra. Acid-fast staining and TB DNA and mycobacterium DNA tests yielded negative results in the aforementioned specimens. Bacterial, mycobacterial, and fungal cultures of the aforementioned specimens yielded negative results as well.

**Fig. 2:**
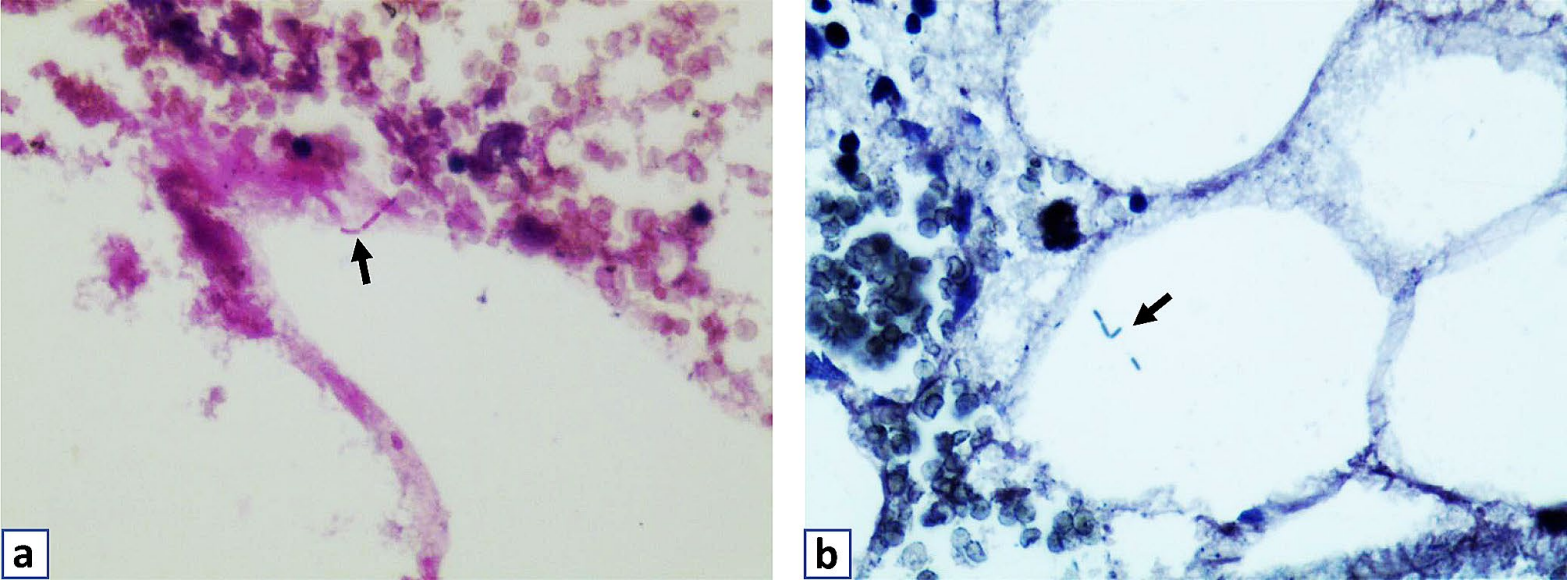
PASM and PAS staining. (**a**) PASM staining showed that the presence of suspected fungal mycelium (black arrows) in the vertebral lesion tissue. (**b**) A suspected single branching hyphae (black arrows) are visible in PAS staining

**Fig. 3:**
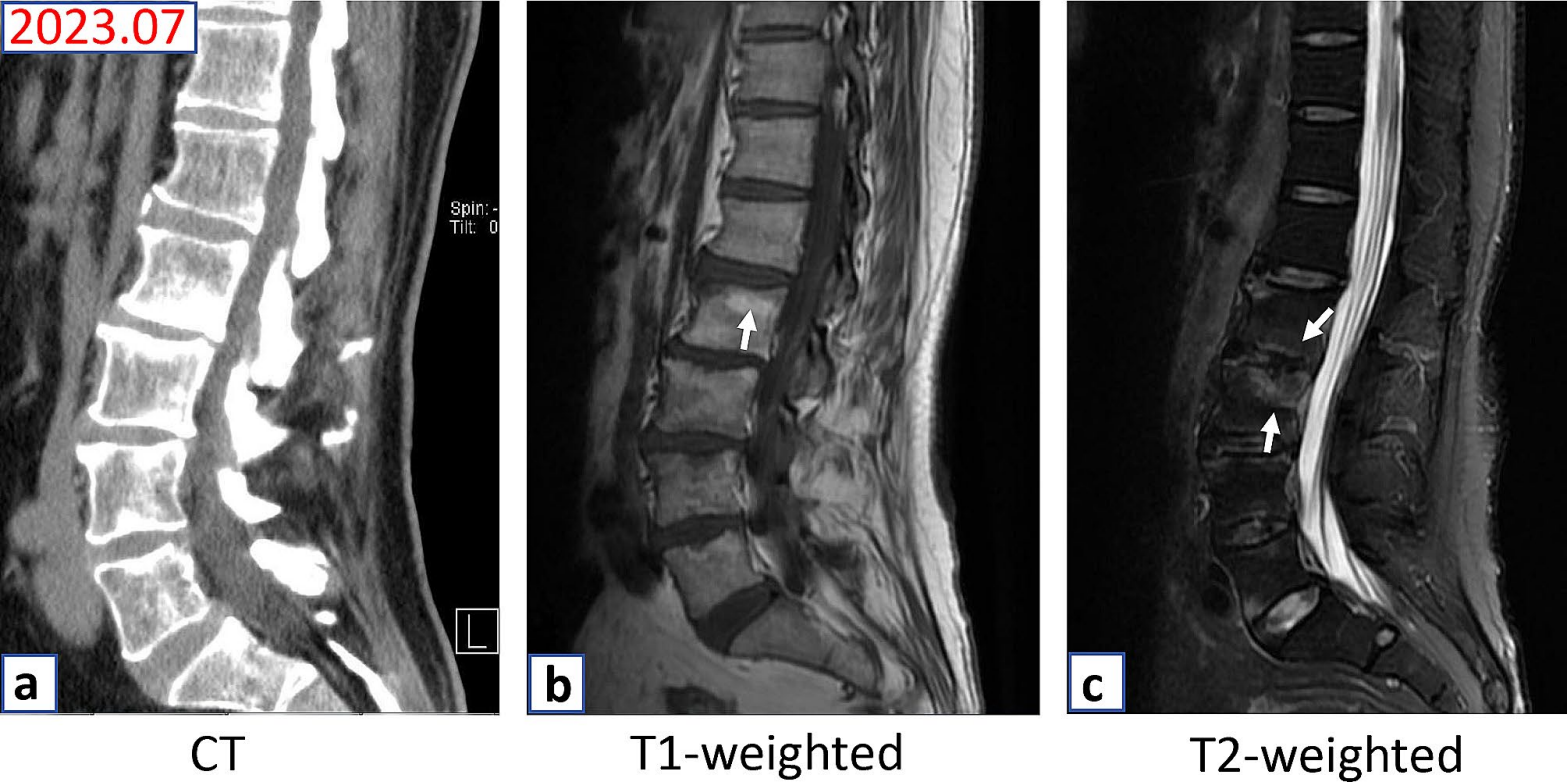
Imaging manifestations after treated with anti-fungal therapy. CT showed an significant reduction of bone destruction in vertebral body (**a**). MRI showed reduced bone destruction at the L2 and L3 vertebral body (white arrow) compared with the imaging performance on July, 2022 (**b&c**)

To detect all potential pathogens and confirm a diagnosis, concurrent PMSeq of the L4 lesion specimens was performed in the Wuhan BGI clinical laboratory. Specifically, the vertebral lesion sample, ranging from 0.5 to 3 mL, was collected from the patient following standardized procedures. Briefly, DNA extraction and construction were performed following the manufacturer’s protocol, which involved DNA fragmentation, end-repair, adapter-ligation, and polymer chain reaction (PCR) amplification. The generation of high-quality sequencing data involved the removal of low-quality reads, followed by computational subtraction of human host sequences that were mapped to the human reference genome (hg19) using Burrows-Wheeler Alignment. After eliminating low complexity reads, the remaining data were classified by simultaneously aligning them to four Microbial Genome Databases, consisting of bacteria, fungi, viruses, and parasites. The classification reference databases were downloaded from the National Center for Biotechnology Information (NCBI) (ftp://ftp.ncbi.nlm.nih.gov/genomes/). Ultimately, the detection of pathogens was reported in accordance with the described criteria [14]. On July 25, 2022, PMseq identified *M. restricta* as the predominant pathogen with the highest relative abundance value (91.08%); the other pathogens identified were *Cutibacterium acnes* (relative abundance value: 21.22%), *Kocuria palustris* (relative abundance value: 5.12%), *Acinetobacter junii* (relative abundance value: 3.55%) and *Pseudomonas monteilii* (relative abundance value: 2.49%). However, *M. tuberculosis* complex was not identified (Table 1). Additionally, we conducted a pathological examination that revealed the presence of fungal mycelium in consecutive sections of the vertebral lesion tissue through periodic Schiff-methenamine (PASM) and periodic acid-Schiff (PAS) staining (Fig. 2). These results suggested that the fungal infection of *M. restricta* was implicated in vertebral body bone destruction, rather than this being a case of tuberculous spondylitis. Therefore, the anti-TB treatment was discontinued, and a combination of fluconazole and voriconazole was administered for antifungal therapy. After a 4-month course of antifungal therapy (4 weeks of oral fluconazole 400 mg/day, then 3 months of oral voriconazole, 300 mg/day), all his symptoms resolved. At the 13-month follow-up, no significant signs of low back pain were evident (VAS score of 1 out of 10), independent walking was achieved, and the range of vertebral lesions was reduced as evidenced by MRI (Fig. 3).

**Table 1:** Results of high-throughput gene detection of PMseq pathogenic microorganisms

Microorganisms category	Type		Genus			Species			Relative abundance	Hierarchy of evidence
			Name	Sequencing reads		Name	Sequencing reads			
Bacteria	-		-	-		-	-		-	Not Detected
Fungi	-		-	-		-	-		-	Not Detected
DNA virus	-		-	-		-	-		-	Not Detected
Parasites	-		-	-		-	-		-	Not Detected
Chlamydia/Mycoplasma/Rickettsia	-		-	-		-	-		-	Not Detected
Background microorganism	G^+^		*Cutibac-terium*	22		*Cutibacerium acnes*	19		21.22%	Suspected
	G^−^		*Pseudo-monas*	10		*Pseudomonas monteilii*	3		2.49%	Suspected
	G^−^		*Acineto-bacter*	9		*Acinetobacter junii*	4		3.55%	Suspected
	G^+^		*Kocuria*	6		*kocuriapalustris*	6		5.12%	Suspected
	Fungi		*Malassezia*	29		*Malassezia restricta*	27		91.08%	Suspected

## Discussion and conclusions

Fungi have been widely acknowledged as an opportunistic causative agent of vertebral osteomyelitis, accounting for 0.5 − 1.6% of spinal infections. The most common etiological agents include *Aspergillus*, *Candida*, and *Mucor* [15, 16]. Patients with fungal spondylitis often present with a high-risk history of fungal infections or comorbidities such as diabetes, leukemia, tumors, and immunocompromised-related diseases [17]. The predominant clinical manifestations of fungal spinal osteomyelitis include local pain, pyrexia, muscular debility, and additional indications of spinal cord compression [18, 19]. Findings on imaging typically resemble those of spinal tuberculosis or spinal tumors, characterized by irregular osteolytic destruction of the vertebral bodies, with or without paravertebral abscesses [20]. After reviewing all English language reports of fungal osteomyelitis involving the spine since 2010, a minimum of 40 related cases were identified (Table 2). Among them, infections included 19 (47.5%) cases of *Candida* [21–37], 10 (25.0%) of *Aspergillus* [21, 22, 29, 38–44], 6 (15%) of *Cryptococcus* [45–50], 2 (5%) cases of *Blastomyces* [51, 52], and 4 (10.0%) cases involving other microorganisms [22, 53–55]. *M. restricta*, an opportunistic fungal pathogen, is implicated in various human skin disorders including tinea versicolor, seborrheic dermatitis/dandruff, atopic dermatitis, folliculitis, and psoriasis [56–58]. However, *Malassezia* has rarely been identified as a pathogen involved in spinal infections, with its main association being chronic infections such as Crohn’s disease, cytokine release syndrome, and respiratory diseases except for skin disorders [7]. Upon retrospective analysis of all previous cases since 2010, we have identified only eight documented instances of *Malassezia* infection, including four patients with endocardial infection [59, 60], three patients with pulmonary infection [61, 62], one patient with liver infection [63], and one patient with synovial infection [64] (Table 3). However, no reports of spinal infections caused by *Malassezia* were found.

In the aforementioned literature review, fungal spondylitis is typically diagnosed through positive cultures and histologic examination of infectious lesion specimens in conjunction with clinical symptoms, laboratory findings, and radiological features. However, our patient was a middle-aged man without any underlying medical conditions or history of abnormal immune function and belonging to an unsusceptible population. The diagnosis of spinal TB is primarily based on the involvement of multiple vertebral bodies, resulting in destruction of the spinal structure observed on CT imaging; however, in this case, evidence from skin, blood tests, or tissue cultures was insufficient to definitively support the presence of infection. The initial pathological examination, including hematoxylin & eosin staining and tissue culture, did not detect any pathogens. Furthermore, the subsequent treatment of anti-TB medication was deemed ineffective. However, it is worth noting that the puncture biopsy under the guidance of CT is recognized as a critical method of diagnosis in cases of unknown etiology of vertebral body destruction [65]. Therefore, PMseq of specimens of the L4 vertebral lesion was performed concurrently to detect all possible pathogens. The results indicated that a fungal infection caused by *M. restricta* may be responsible for the patient’s vertebral body destruction, rather than being a case of tuberculous spondylitis. Metagenomic sequencing, as an innovative culture-independent approach, possesses the capacity for rapid, sensitive, and accurate identification of pathogens. Numerous recent studies have demonstrated that metagenomic sequencing plays a pivotal role in successful pathogen recognition for infections characterized by atypical clinical symptoms that are unidentifiable through traditional etiological detection methods or caused by unknown pathogens [66, 67]. These findings underscore the pivotal role of metagenomic sequencing in guiding drug administration and enhancing clinical outcomes for patients with spinal infection, particularly when conventional methods fail to identify the pathogen [68]. In this study, the pathological examination revealed the presence of fungal mycelium in serial sections of the vertebral lesion tissue, as indicated by positive results of PASM and PAS staining. Consequently, the anti-TB treatment was replaced with a combination of ketoconazole and itraconazole as antifungal therapy, which is widely acknowledged as essential and efficacious for managing fungal osteomyelitis [69]. In our review, a favorable outcome was observed in 9 out of 40 cases (22.5%) who received prolonged treatment with antifungal agents. Notably, early identification and sustained administration of appropriate antifungal drugs such as fluconazole, voriconazole, etc., significantly enhanced the prognosis for patients with *M*. infection. Fortunately, the patient’s symptoms of impaired ambulation and lumbago were successfully alleviated based on the VAS score, whereas the extent of vertebral lesions demonstrated a reduction on MRI. Although no precise pathogen evidence was detected through blood tests and tissue culture, the therapy was proved effective for the patient following guidance from PMseq of infected tissue. Furthermore, confirmation of *Malassezia* infection may be achieved through a combination of pathology, blood cultures, and other methods involving selective pathological examination and PCR testing [70]. However, this study is limited by the absence of definitive evidence to ascertain the causative pathogen responsible for the infection in the tissue culture. Additionally, we did not rule out whether the injection of COVID-19 vaccine could be a risk factor for this infection because the time of onset of illness was within 6 months after the injection of the COVID-19 vaccine. Nevertheless, the PMseq results combined with suspected fungal presence were further supported by fungal mycelium observation from PASM and PAS staining, CT imaging, and the efficacy of antifungal treatment. These findings provide additional confirmation to the speculation that *M. restricta* is recognized as the most predominant pathogen .

In summary, we present the first reported case of spondylitis caused by *Malassezia* and provide a comprehensive review of 40 published cases of fungal spondylomyelitis as well as eight cases of systemic organ infection caused by *Malassezia*. Given the atypical clinical symptoms and imaging findings observed in fungal spondylitis, along with the high rates of negative diagnosis for *Malassezia*, a single diagnostic method may not be sufficient to accurately diagnose *Malassezia* spondylitis. Therefore, employing PMseq from affected tissue could serve as a rapid and precise approach to guide drug management and clinical treatment for certain non-specific infectious diseases.

**Table 2:** Main characteristics of spine fungal osteomyelitis

Author	Age/sex	fungal	Clinical presentation	Diagnosis techniques	Treatment	Antifungal drug	Method of medication	Course of medication	Follow up	Clinical outcome
Williams et al. [21]	49/M	*Candida albicans*	progressive pain in the lumbosacral region	biopsy, Culture and sensitivity	L2–L3 and L3–L4 diskectomy and vertebral debridement	unclear	unclear	unclear	unclear	unclear
Williams et al. [21]	51/M	*Candida*	lower back pain	biopsy, histologic examination	Drug	amphotericin, fluconazole	a 6-week course of intravenous amphotericin followed by oral fluconazole	unclear	5 months	Improvement
Williams et al. [21]	54/F	*Aspergillus*	lower back pain	biopsy, histologic examination	diskectomy and vertebral debridement of the L2–L3 and L3–L4 levels and drug	amphotericin.	unclear	unclear	unclear	unclear
Keerthi et al. [22]	37/M	*Aspergillus*	back pain	Histopathological investigations from intra operative	L1-2 Posterior Stabilization, Debridement & Fusion and drug	Voriconazole	Inj.Voriconazole 200 mg Infusion twice daily was given for 5 days and patient was discharged with oral Voriconazole for 3 months.	3 months	3 months	Full recovery
Keerthi et al. [22]	75/M	*Candida albicans.*	back pain	Histopathological investigations from intra operative	Anterior & Posterior Spinal Debridement, Fusion at L2-3 and drugs	caspofungin, fluconazole	Injection caspofungin was given for 7 days and patient was discharged with oral fluconazole for 6 months.	6 months	6 months	Full recovery
Keerthi et al. [22]	56/M	*Scedosporium apiospermum*	back pain	Histopathological investigations from intra operative	D5-11 Segmental Posterior Stabilization, Decompression &Fusion and drugs	Voriconazole	Voriconazole 200 mg infusion over 2 h twice daily for 5 days, discharged with oral Voriconazole 200 mg twice daily	1 months	1 months	diead
Gagliano et al. [23]	66/M	*C. glabrata*	ward for lumbar pain, progressive difficulty in walking, fever	open biopsy of an abscess and culture examination	debridement and stabilization of the vertebrae involved and drug	anidulafungin	anidulafungin 200 mg on the first day, followed by 100 mg daily thereafter	unclear	unclear	Full recovery
Overgaauw et al. [24]	78/M	*C. krusei*	lower back pain	Histopathological investigations from biopsy	operative and drugs	anidulafungin, voriconazole, amphotericin B	anidulafungin (loading dose of 200 mg IV, followed by 100 mg/day IV) oral voriconazole (200 mg/twice daily) amphotericin B (3 mg/kg)	9 months	9 months	Improvement
Yu et al. [25]	76/M	*Candida albicans*	back pain and fever	Histopathological investigations from intra operative	operative and drugs	fluconazole	fluconazole intravenously for 1 month and then orally for 3 months.	4 months	12 months	Improvement
Kelesidis and Tsiodras [26]	41/M	*Candida*	low back pain	biopsy, histologic examination	drug	caspofungin	high-dose caspofungin 100 mg IV daily for 6 weeks	6 weeks	10 months	Full recovery
Eda et al. [27]	70/M	*Candida*	low back pain fever	culture examination from intra operative	operative and drugs	fluconazole	intravenous fluconazole (800 mg/day) three weeks, followed by an oral administration of fluconazole (400 mg/day)	unclear	unclear	Improvement
Cevolani et al. [28]	57/M	*C. tropicalis*	persistent low back and leg pain	biopsy, histologic examination	drug	fluconazole	fluconazole (6 mg/kg/day)	unclear	12 months	Improvement
Adhikari et al. [29]	64/M	*Aspergillus, C. albicans*	pain in bilateral, fever	culture examination from intra operative	operative and drugs	fluconazole	fluconazole for 12months	unclear	unclear	Improvement
Wang. et al. [30]	62/F	*Candida tropicalis*	low back pain, fever	culture examination from endoscopic discectomy operative	operative and drugs	amphotericin B	unclear	unclear	unclear	Recurrence and reoperation
Gopinathan et al. [31]	19/F	*Candida tropicalis*	back pain	culture examination from endoscopic discectomy operative	operative and drugs	amphotericin B	Inj Amphotericin B for 14 days followed by oral fluconazole 2 for three months.	3 months	unclear	Improvement
Gopinathan et al. [31]	64/M	*Candida tropicalis*	back pain	culture examination from endoscopic discectomy operative	operative and drugs	fluconazole	oral fluconazole twice a day and then continued with fluconazole once daily for 6 weeks	6 weeks	6 months	Improvement
Yamada et al. [32]	74/M	*Candia albicans*	low back pain, right leg pain and gait disturbance	Histopathological investigations from intra operative	operative and drugs	fluconazole	fluconazole intravenously per day, for 2 weeks, later changed to fluconazole	3 months	9 months	Full recovery
Tan et al. [33]	47/M	*Candida glabrata*	back pain	unclear	drug	fluconazole, caspofungin, posaconazole	unclear	unclear	unclear	Improvement
Nahra et al. [34]	31/F	*Candida albicans*	back pain	culture examination from biopsy	operative and drugs	micafungin, fluconazole	fluconazole 400 mg for 6 months	6 months	12 months	Improvement
Kulcheski et al. [35]	39/M	*Candida albicans*	paralyzed	culture examination	operative and drugs	fluconazole and Ciprofloxacin	unclear	unclear	unclear	Improvement
Koehler et al. [36]	56/M	*Candida*	back pain	culture examination from biopsy, assay measuring Candida reactive CD4+ T cells	operative and drugs	caspofungin	caspofungin 70/50 mg was initiated for 8 weeks	unclear	unclear	Full recovery
Huang et al. [37]	32/M	*Candida albicans*	progressive limited cervical range of motion, neck pain, bilateral upper extremity weakness, and paresthesias	culture examination from operative	operative and drugs	fluconazole	6–12 months of 400 mg fluconazole	6–12 months	3 months	Improvement
Shashidhar et al. [38].	33/F	*Aspergillus fumigatu*	low back pain	culture examination from intra operative	operative and drugs	voriconazole	i.v. voriconazole 200 mg twice daily for one week followed by oral voriconazole 200 mg twice daily for 11 weeks.	3 months	12 months	Full recovery
Karthik et al. [39]	13/M	*aspergillus*	gradual onset paraplegia	Histopathological investigations from intra operative	operative and drugs	amphotericin B, voriconazole	intravenous amphotericin B intravenous voriconazole for 2 months followed by oral voriconazole	24months	24months	Improvement
Jiang et al. [40]	40/F	*Aspergillus nidulans*	back pain, numbness and weakness of both lower limbs, A CT-guided needle biopsy of the paravertebral lesion, fungal culture and PCR analysis	culture examination from operative	operative and drugs	fluconazole	voriconazole every 12 h as a loading dose on day 1, followed by voriconazole twice daily, voriconazole every 12 h administered orally for 6 months	6 months	28 months	Full recovery
Sohn .et al. [41]	12/F	*Aspergillus terreus*	back pain	biopsy, histologic examination	drug	voriconazole	IV voriconazole (5 mg/kg, q12hr) for 4 and 8 weeks, oral voriconazole (200 mg [5 mg/kg/dose], bid) for 9 months.	9 months	23 months	Improvement
Yoon and Kim [42].	53/M	*Aspergillus*	back pain	Histopathological investigations from intra operative	operative and drugs	amphotericin B	unclear	unclear	unclear	Improvement
Li et al. [43]	51/M	*Aspergillus*	cough, fever, and low back pain	Histopathological investigations from intra operative	operative and drugs	voriconazole	a loading dose of 6 mg/kg of body weight on the 1st day followed by 4 mg/kg, every 12 h intravenously	3 months	3 months	Improvement
Al-Tawfiq et al. [44]	17/M	*Aspergillus*	back and neck pain, fever	biopsy of an abscess and culture examination	operative and drugs	amphotericin-B, itraconazole, voriconazole.et.al.	unclear	more than 24 months	more than 5 years	Improvement
Lai et al. [45]	25/M	*cryptococcal*	back pain	Histopathological investigations from intra operative	operative and drugs	amphotericin B	4 weeks of intravenous amphotericin B and then 8 weeks of oral amphotericin B	3 months	9 months	Improvement
Zhong et al. [46]	37/M	*Cryptococcus neoformans*,	progressive low-back and sacrococcygeal-pain	biopsy of an abscess and culture examination	drug	fluconazole	4 weeks of intravenous fluconazole, then 8 weeks of oral fluconazole	3 months	8 months	Full recovery
Adsu et al. [47]	45/F	*cryptococcal*	back pain	Histopathological investigations from intra operative	operative and drugs	fluconazole, amphotericin B	amphotericin B ntravenously for 3 months, oral fluconazole 400 mg once a day for 5 months.	8 months	unclear	Improvement
Li .et al. [48]	17/F	*cryptococcal*	back pain, fever, scoliosis	Histopathological investigations from intra operative	operative and drugs	fluconazole	fluconazole for 3months	3 months	unclear	Improvement
Pereira-Duarte et al. [49]	65/M	*cryptococosis*	back pain	biopsy, histologic examination	operative and drugs	fluconazole, amphotericin B	unclear	12 months	12 months	Improvement
Nankeu et al. [50]	29/M	*cryptococcosis*	back pain, fever,	biopsy, histologic examination	drug	amphotericin B, flucytosine, fluconazole	amphotericin B and flucytosine for 4 weeks followed by fluconazole for 18 months	19 months	unclear	Improvement
Eldaabossi et al. [51]	24/M	*Blastomyces*	superficial abscess, dry cough, fatigue, and profuse sweating	Histological cytology after multiple biopsies	Abscess drainage and drug	itraconazole	itraconazole 200 mg orally twice daily for 12 months	12 months	12 months	Full recovery
Sapra et al. [52]	42/M	*Blastomyces*	knee pain	culture examination from operative	drug	amphotericin B, itraconazole	a four-week course of IV amphotericin B and then start on a six-month regimen of oral itraconazole	6 months	6 months	Improvement
Alvarenga et al. [53]	68/M	*Paracoccidioides * *brasiliensis*	low-back pain	percutaneous biopsy	Drug	itraconazolesulfamethoxazole and trimethoprim	Treatment with itraconazole (200 mg/day) resulted in adverse reactions and switching to sulfamethoxazole and trimethoprim (20 mg/kg) for 36 months	36 months	36 months	Improvement
Blecher et al. [54]	67/M	unclear	back pain	Histopathological investigations from intra operative	operative and drugs	fluconazole	unclear	unclear	unclear	Improvement
Shimizu et al. [55]	45/M	*S. apiospermum*	back pain	culture examination from endoscopic discectomy operative	operative and drugs	itraconazole	200 mg of itraconazole intravenously administered once daily for 8 weeks, oral dose of 100 mg of itraconazole was given for 12 months	14 months	20 months	Full recovery

**Table 3:** Main characteristics of spine fugal osteomyelitis

Infected site	Clinical symptoms	Past history	Detection method	Bacteria of infection	Treatment
Endocarditis [59]	febrile, and cardiac auscultation noted a soft holosystolic murmur	injection drug use, surgical history	Fungiculture and Blood cultures	*Malassezia furfur*	Voriconazole (Recovery)
Endocarditis [60]	Acute cardiac failure	Itching cutaneous lesions and undergone cardiac valvular prosthesis implantation	GrocottGomori, Staining Autoimmunohisto-chemistry,18–26 S-rDNA and ITS PCRs	*Malassezia restricta*	None (death in heart failure)
Endocarditis [60]	Acute cardiac failure Multiple septic emboli	Itching cutaneous lesions and undergone cardiac valvular prosthesis implantation	GrocottGomori, Staining Autoimmunohisto-chemistry,18–26 S-rDNA and ITS PCRs	*Malassezia restricta*	Caspofungin(death in iterative nondocumented sepsis)
Endocarditis [60]	Acute cardiac failure and Multiple septic emboli	Itching cutaneous lesions and undergone cardiac valvular prosthesis implantation	Grocott-Gomori, Staining Auto-immunohisto-chemistry,18–26 S-rDNA and ITS PCRs	*Malassezia restricta*	Amphotericin B fluorocytosin and fluconazole (Recovery)
Fungemia infection and lung emboli [61]	Cough, Lung Nodules, Fever, and Eosinophilia	Unknown	blood culture for plating on olive oil, Giemsa stain and DNA sequencing	*Malassezia sympodialis*	liposomal amphotericin B (Recovery)
Pneumonia [62]	fever	double-lung transplant	PAS staining and PCR 18 S-sequencing	*Malassezia restricta*	Voriconazole(Recovery)
Pneumonia [62]	pulmonary nodule	kidney transplant	PAS and Grocott staining and PCR 18 S-sequencing	*Malassezia restricta*	Itraconazole(Recovery)
Hepatic Abscess [63]	fever and a liver mass	Neonate	Gram stain, aerobic and anaerobic bacterial culture, and acid-fast bacilli stain and culture and Calcofluor white stain	Unknown	amphotericin B(Recovery)
Arthritis [64]	pain, swelling, erythema and fever	total knee arthroplasty surgeryskin disease	Gram-staining	Unknown	amphotericin B, fluconazole and Voriconazole(Recovery)

## Data Availability

The datasets generated and analyzed during the present study are available from the corresponding author on reasonable request.

## Abbreviations

TB: Tuberculosis
M: Restricta, Malassezia. Restricta
PMseq: Pathogen Metagenomics Sequencing
MRI: Magnetic Resonance Imaging
PASM: Periodic-acid silver methenamine
PAS: Periodic acid shiff
VAS: Visual Analogue Scale
CT: Computed Tomography
